# Mammal road‐type associations in Kruger National Park, South Africa: Common mammals do not avoid tar roads more than dirt roads

**DOI:** 10.1002/ece3.8190

**Published:** 2021-10-26

**Authors:** Misha Malherbe, Trevor McIntyre, Tarryn V. Hattingh, Paige M. Leresche, Natalie S. Haussmann

**Affiliations:** ^1^ Department of Geography, Geoinformatics and Meteorology University of Pretoria Hatfield South Africa; ^2^ Department of Life and Consumer Sciences University of South Africa Florida South Africa

**Keywords:** *Aepyceros melampus*, *Equus quagga*, habituation, *Loxodonta africana*, road avoidance, roadside ecology

## Abstract

The majority of Africa's parks and conservation areas have a vast road network, facilitating motorized vehicle game viewing. These roads have an influence that is both road type‐ and species‐specific, on the surrounding ecosystem. Due to their higher traffic volumes, we hypothesized that tar roads and their immediate surrounds within the Kruger National Park, South Africa, are avoided to a greater extent by medium‐to‐large mammals than comparable dirt roads in the park. We systematically recorded the presence of medium‐to‐large mammal species from our vehicle, recording data at 401 tar and 369 dirt road stops in the Kruger National Park. In addition to species presence, we also estimated the proximity of animals to the road, as well as herd sizes. Our results indicate an equal likelihood of viewing the commonly recorded medium‐to‐large mammal species from both road types. The likelihood of observing larger herd sizes was also similar between tar and dirt roads for the three most commonly observed species, African elephant (*Loxodonta africana*), impala (*Aepyceros melampus*), and plains zebra (*Equus quagga*), and the likelihood of viewing impala and zebra close to the road also did not differ between tar and dirt roads. However, elephant was observed more often close to tar roads, compared to dirt roads. We interpreted this as the result of potentially increased woody cover associated with more water runoff in close proximity to tar roads compared with dirt roads. Our results not only have ecological significance, supporting the notion that many of the park's species are habituated to human infrastructure, but also management implications, informing park officials about the influence of road traffic and road type on wildlife distributions.

## Graphical abstract

The likelihood of viewing the common mammal species in the Kruger National Park (KNP), South Africa, is equal from tar and dirt roads. The likelihood of viewing large herds of elephant (*Loxodonta africana*), impala (*Aepyceros melampus*), and zebra (*Equus quagga*) is equal from tar and dirt roads in KNP. The likelihood of close‐up sightings of impala and zebra is equal from tar and dirt roads, but close‐up elephant sightings are more likely next to tar than dirt roads at KNP.

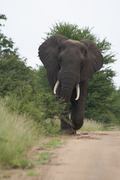

## 1 INTRODUCTION

Roads have ecological impacts on the natural areas surrounding them—areas that are also referred to as “road effect zones” (Forman & Alexander, 1998). Road impacts on wildlife typically happen through three mechanisms: Roads decrease habitat quality, they decrease the connectivity of the landscape, and they potentially decrease population survival by increasing mortality rates (roadkill) (Teixeira et al., 2020). While it is not always known exactly which of these three pathways—or a combination of these—is the cause (Teixeira et al., 2020), records of negative responses of wildlife to roads are plentiful (see Benítez‐lópez et al., 2010; Fahrig & Rytwinski, 2009; Forman & Alexander, 1998 for reviews).

Road construction and use can lead to an increase in noise exposure, which results in both cumulative and secondary impacts, such as habitat avoidance by animals surrounding high traffic density road networks (Benítez‐López et al., 2010). Such habitat degradation as a result of roads can cause a change in the structural and functional links among the ecosystem relationships, resulting in a biodiversity change (Evink & Erickson, 2002). Roads can also act as barriers, prohibiting or limiting the movement of species between habitats or between populations (McGregor et al., 2008; Newmark, 2008). This leads to habitat and population fragmentation (Millions & Swanson, 2007) and the isolation of animal species (Saunders et al., 2002), or even the localized extinction of populations (Leblond et al., 2013).

The extent of road influence is often species‐specific (Benítez‐López et al., 2010), with some species even experiencing roads as beneficial (May & Norton, 1996). Such species actively make use of roads, with the most common reasons being foraging, easier movement, and refuge (Hill et al., 2021). For example, moose, *Alces alces*, in the Greater Yellowstone Ecosystem utilize road verges surrounding high traffic density roads to avoid predation from road averse predators, such as brown bears, *Ursus arctos* (Berger, 2007). In addition, some predators, such as red foxes, *Vulpes vulpes*, use road networks for traveling (Ramp et al., 2006), as roads provide easy traveling conditions (Hill et al., 2021; May & Norton, 1996). However, other predators, such as Scandinavian wolves, *Canis lupus* and grizzly bears, *Ursus arctos*, tend to avoid road environments (Ciarniello et al., 2006; Karlsson et al., 2007). Furthermore, large mammals are more likely to be negatively affected by roads than smaller species, because they have longer life cycles and lower reproduction rates and are therefore less able to recover rapidly from population declines due to road mortality (Rytwinski & Fahrig, 2011). Some evidence exists also for large African mammals avoiding roads. For example, African elephant, *Loxodonta africana*, and eland, *Taurotragus oryx* show a decrease in density in a zone up to 600 m from road networks (Newmark et al., 1996). Small mammals, in contrast, often come close to roads (Benítez‐López et al., 2010; Ramp et al., 2006) and some species, such as white‐footed mice, *Peromyscus leucopus* and eastern chipmunks, *Tamias striatus*, are seemingly unaffected by roads (McGregor et al., 2008).

In addition to road impacts being species‐specific, they also appear to be road type‐specific, with tar (paved) roads possibly having a greater impact on the ecosystem than dirt (unpaved) roads (van der Ree et al., 2015). For example, in the Kruger National Park, South Africa, reduced presence and increased flight response of impala, *Aepyceros melampus*, was associated with tar roads, compared to dirt roads (Mulero‐Pázmány et al., 2016, but also see Jackson et al., 2017). One of the potential reasons for these differences in ecological impacts between tar and dirt roads is the difference in traffic intensity, as some species tend to avoid roads with greater disturbance intensity (Fahrig & Rytwinski, 2009). For example, forest‐dwelling caribou, *Rangifer tarandus*, are more likely to avoid the 5 km road effect zone when there are higher traffic volumes (Leblond et al., 2013) and male red deer, *Cervus elaphus*, in the USA establish seasonal home ranges closer to quiet roads, where they are subsequently joined by herds of females (Montgomery et al., 2013). However, evidence to the contrary also exists, even within the same species. Road avoidance by red deer in Norway (Meisingnet et al., 2013) and Spain (D'Amico et al., 2016) is similar between road types, irrespective of the surface type, and associated traffic volumes.

In South Africa, many of the nature reserves have roads that experience regular traffic, mostly for game viewing by tourists. In addition to the tourist traffic within South Africa's parks, some parks are also crossed for daily transportation. Most parks within South Africa have residential and developed areas situated on the periphery. As a result, both commuters and goods are often transported through the parks. For example, in the Kruger National Park, mini buses regularly use park roads to transport commuters from the adjacent areas through the reserve, as this is often the shortest route (Connor, 2003). In addition, goods are transported daily by means of heavy vehicles to lodges and camps within the park, increasing the traffic density of the parks' roads (Rogerson, 2012).

The aim of this study was to compare potential road avoidance of medium‐to‐large mammal species between tar and dirt roads in the Kruger National Park. Greater road avoidance was expected for tar roads compared with dirt roads, as the tar roads of the park have a higher traffic volume than its dirt roads (Mulero‐Pázmány et al., 2016). Three hypotheses were formulated, based on this increased traffic volume associated with tar roads. First, we hypothesized that we would record fewer mammal sightings next to tar than dirt roads. Next, we hypothesized that avoidance of tar roads would result in smaller mammal herd sizes next to tar than dirt roads. And lastly, we hypothesized that tar road avoidance would result in fewer sightings in close proximity to tar than dirt roads.

## 2 MATERIALS AND METHODS

### 2.1 Study area

The Kruger National Park is the largest game reserve in South Africa, comprising of almost 2 million hectares of government‐managed land. The park has also recently removed fences between Mozambique and Zimbabwe to create the Greater Limpopo Transfrontier Park, which allows for an open wilderness area between the three countries. The climate ranges from tropical to subtropical, and rainfall mostly falls in the form of summer thunderstorms between November and March. The park is characterized by a rainfall gradient from south to north (the south experiencing approximately 590 mm annual rainfall on average and the north experiencing approximately 470 mm annual rainfall on average). Summers (October to March) in the park are predominantly hot and rainy (mean daily temperature range = 18–31℃), while winter months (April to September) are usually dry, with warm days and cold nights (mean daily temperature range = 13–28℃) (South African Weather Services, unpublished data).

The Kruger National Park hosts 21 vegetation types, of which savanna systems dominate (Mucina & Rutherford, 2006). More than 150 species of mammal have been recorded in the park (Spies et al., 2018). In total, the 2294 km of public roads in the park consists of 850 km tarred road and 1444 km dirt road, with the majority of the main routes between camps consisting of tar roads (Joubert, 1990). On average, the traffic volume is six times greater on tar roads than dirt roads (Mulero‐Pázmány et al., 2016).

### 2.2 Data collection

Fieldwork was carried out during two field sessions, one in 2017 and one in 2019. Although there was a two‐year gap between the field sessions, both sessions happened in July, during the Austral winter. In addition, neither rainfall nor temperature data differed substantially between these years (*t* test, rainfall: *t* = 1, *n* = 31, *p* = .16; temperature: *t* = −0.70, *n* = 62, *p* = .24) (data from South African Weather Services, unpublished data). Visitor numbers to the park were also generally similar between 12‐month periods that spanned the field sessions (April 2017–March 2018 = 1,932,750; April 2018–March 2019 = 1,892,128; April 2019–March 2020 = 1,833,061) (South African National Parks, 2018, 2019, 2020), and no other factors that could influence animal behavior toward road type were considered likely to have differed substantially between field sessions.

**FIGURE 1:**
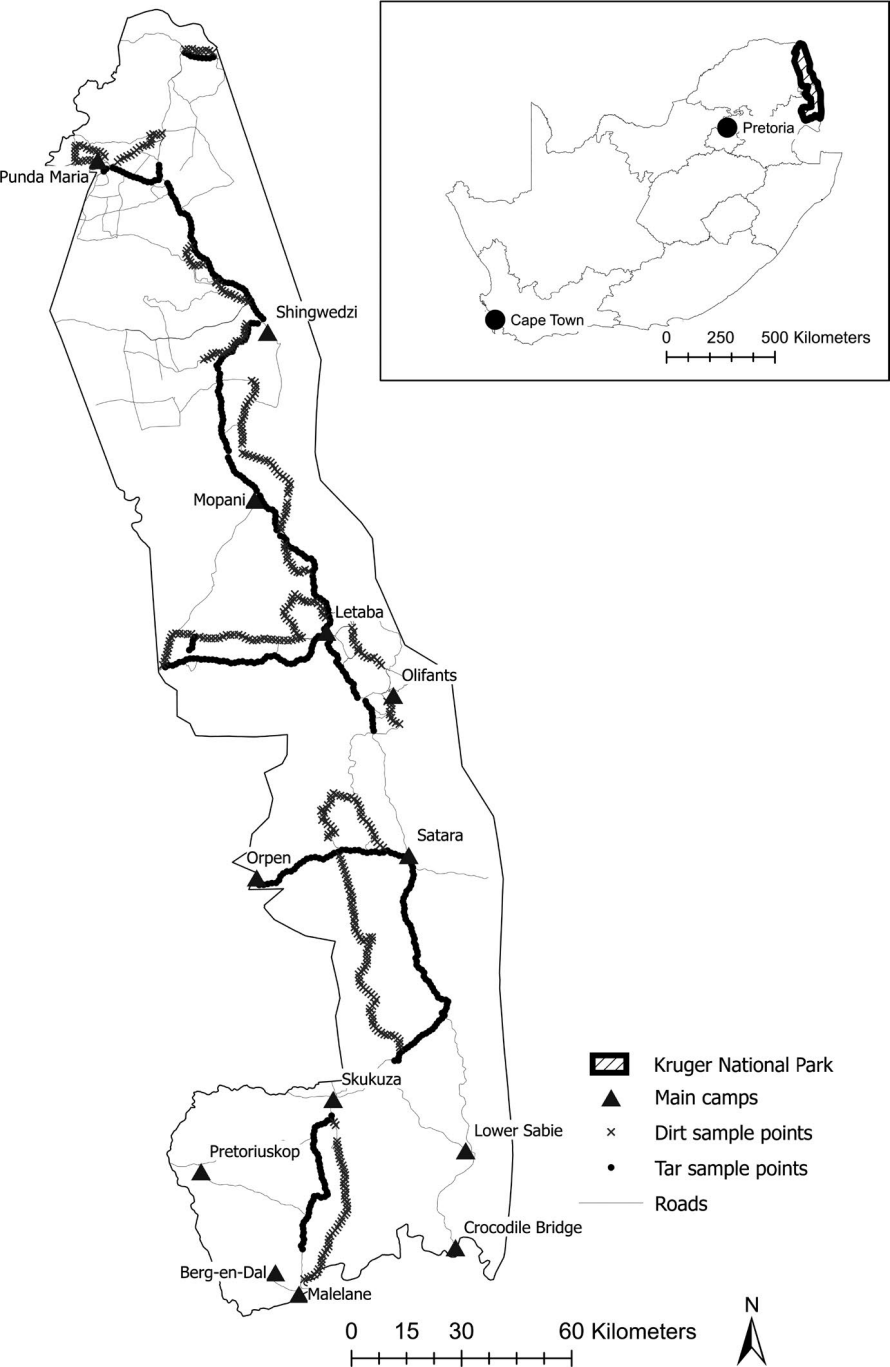
Tar and dirt road sampling points in the Kruger National Park

Two observers systematically recorded the presence of medium‐sized to large mammals, stopping every 1 km along tar and dirt roads, between 7h30 and 15h00. To control for the influence of the time of day, we alternated sampling on tar roads and dirt roads on a daily basis. Thus, we sampled one whole day on tar and the next whole day on dirt. Alternating daily also minimized weather influences, which might change over the course of the season. The main tar roads, connecting the main rest camps, were sampled, and equivalent distances of dirt road that ran, as far as possible, parallel to the tar road, were selected (Figure 1). We took care to avoid dirt road loops, which often lead to watering holes. This ensured that there was no association between road type and distance to natural water sources (ANOVA, *f* = 0.04, *df* = 1, *p* = .85). Furthermore, none of the roads that we sampled on crossed through any of the rest camps or other human settlements. We also only recorded in the dominant vegetation type, namely savanna, as other vegetation types (e.g., forest and azonal vegetation) were too dense to sight animals more than a few meters from the vehicle. In general, savanna vegetation consists of open landscapes with good visibility, and, within savanna vegetation, there was no noticeable difference in the range of sight between tar and dirt roads. In total, 401 tar road records and 369 dirt road records were obtained over a total period of two months.

At each stop, we recorded all observed medium‐to‐large mammal species during a 10‐min interval. We then categorized the distance of the closest individual of each species to the road into one of two distance categories—close (<20 m from the road) and far (>20 m from the road). Distance estimation was practiced beforehand at the campsite using a measuring tape. All visible individuals were counted when fewer than 30 were visible, while larger group/herd sizes (>30) were estimated to the nearest interval of 10 animals based on sub‐counts of groups of 10 individuals and extrapolating across individuals visible. We subsequently categorized group/herd size into one of two categories (<6 individuals and >=6 individuals), based on an evident binomial distribution in group/herd sizes. We also recorded the traffic volume as the number of vehicles passing us from either direction during the 10‐min interval. Lastly, we recorded the GPS coordinate of the stop (Garmin GPSMAP 62s) and we estimated the percentage cloud cover. Afterward, distances between sampling points and nearest natural water source were measured using ArcGIS 10.8 (ESRI, Inc.). Artificial water sources are not static, and we ignored these in this study since their locations and status (open/closed) could not be determined.

### 2.3 Data analyses

All statistical analyses were performed in the R programming environment (R Core Team, 2020). Chi‐square tests of association were performed to identify potential associations between species presence and road type. Seven species occurred often enough for these tests to be statistically meaningful (i.e., expected presences >5, Larntz, 1978). These were as follows: buffalo (*Syncerus caffer*), elephant (*Loxodonta africana*), giraffe (*Giraffa camelopardalis*), impala (*Aepyceros melampus*), kudu (*Tragelaphus strepsiceros*), warthog (*Phacochoerus africanus*), and zebra (*Equus quagga*).

Generalized linear models (GLZs) were used to model the relationships between: (a) herd size (response variable) and road type (explanatory variable), as well as (b) the distances that animals were from the road when observed (response variable) and road type (explanatory variable). These analyses were conducted only for those species with more than ten sightings per road type (elephant: 22 tar, 14 dirt, impala: 53 tar, 61 dirt and zebra: 19 tar and 22 dirt). Since distance to road and herd size were converted to two categories each, a binomial distribution was assumed for these variables. In addition, distance to the nearest water source and cloud cover were added as covariates for both response variables. However, traffic volume was strongly related to road type, with tar roads having significantly higher traffic volumes than dirt roads (Mann–Whitney *U* test, *p* < .01). We therefore did not include traffic volume in any of the models. All possible combinations of fixed variables were then compared in order to select the most parsimonious models using the “dredge” function in the *MuMIn* package (Barton, 2020). Model selection was undertaken based on maximum likelihood and using second‐order AIC (AICc) scores and corresponding AIC weights to select the most parsimonious models (Burnham & Anderson, 2002). Statistical significance was set at *p* ≤ .05.

## 3 RESULTS

We found a five times higher traffic volume on the tar roads (0.48 ± 0.33 vehicles/min) than the dirt roads (0.10 ± 0.13 vehicles/min) of the Kruger National Park. In total, the seven most often‐observed species were observed at 95 tar and 103 dirt road stops. With 114 sightings in total, impala were observed most often (Table 1), followed by zebra (41 sightings), elephant (36 sightings), kudu (19 sightings), giraffe and warthog (17 sightings each), and buffalo (11 sightings). None of the seven species showed a significant association with road type (Table 1), and thus, one's chances of viewing any of these species from either road type are equal. The largest herd sizes were seen for buffalo and impala, with estimated herd sizes of up to 200 individuals observed. Other species that were observed in group sizes of more than 30 individuals were elephant and zebra. Observations of all seven often‐observed species included sightings of individuals on the road for both road types. For elephant, impala, and zebra, best models for both herd size and distance to road included all three explanatory variables (Table 2). However, only road type had a significant effect, specifically on the observation distances of elephant. Thus, closer sightings of elephant are more likely next to tar roads (13 out of 22 sightings) than dirt roads (four out of 14 sightings). None of the other explanatory variables contributed significantly to explaining either herd size or distance from road for any of the three species (Table 2).

**TABLE 1:** Results of the chi‐square tests of association with road type for the seven most frequently observed species

Species	Presence		Test output
	Yes	No	
Buffalo			
Tar	5	396	χ^2^ = 0.196, *df* = 1, *p *= .66
Dirt	6	363	
Elephant			
Tar	22	379	χ^2^ = 1.235, *df* = 1, *p *= .27
Dirt	14	355	
Giraffe			
Tar	8	393	χ^2^ = 0.176, *df* = 1, *p *= .68
Dirt	9	360	
Impala			
Tar	53	348	χ^2^ = 1.673, *df* = 1, *p *= .20
Dirt	61	308	
Kudu			
Tar	8	393	χ^2^ = 0.776, *df* = 1, *p *= .38
Dirt	11	358	
Warthog			
Tar	10	391	χ^2^ = 0.317, *df* = 1, *p *= .57
Dirt	7	362	
Zebra			
Tar	19	382	χ^2^ = 0.571, *df* = 1, *p *= .45
Dirt	22	347	

Note

In total, there were 401 tar and 369 dirt road sampling stops. Affirmative presences show the number of these stops at which a species was observed during the 10‐min sampling interval.

Significant differences were taken as *p* < .05.

**TABLE 2:** Generalized linear model (GLZ) outputs for elephant, impala, and zebra herd size and distance to road, showing all fixed effects retained in final models

Species	Response variable	Fixed effects	χ^2^	*df*	*p*
Elephant *n* = 36	Herd size	Cloud cover	0.88	1, 33	.35
		Nearest River	0.95	1, 32	.33
		Road type	0.56	1, 34	.45
	Distance to the road	Cloud cover	1.68	1, 33	.20
		Nearest River	2.79	1, 32	.09
		Road type	4.16	1, 34	.04*
Impala *n* = 114	Herd size	Cloud cover	1.46	1, 11	.23
		Nearest River	0.76	1, 11	.38
		Road type	2.61	1, 11	.11
	Distance to the road	Cloud cover	0.48	1, 11	.49
		Nearest River	1.14	1, 11	.29
		Road type	1.62	1, 11	.20
Zebra *n* = 41	Herd size	Cloud cover	2.75	1, 38	.10
		Nearest River	3.64	1, 37	.06
		Road type	0.04	1, 39	.85
	Distance to the road	Cloud cover	0.03	1, 38	.86
		Nearest River	3.42	1, 37	.06
		Road type	<0.01	1, 39	.97

Note

Final models were selected based on AICc scores (Burnham & Anderson, 2002).

Statistical significance (*) was set at *p* < .05.

## 4 DISCUSSION

Roads can influence herbivores, either positively by creating corridor habitats (van der Ree et al., 2015) and providing access to previously unavailable forage, nutrient, and water resources (Ramp et al., 2006) or negatively by acting as a barrier, hindering, or impeding movement (Newmark et al., 1996), reducing habitat quality (van der Ree et al., 2015), and causing fatal collisions (Ramp et al., 2006). The effect is, however, dependent on the specific species (Ramp et al., 2006), as well as the road type and traffic volume (van der Ree et al., 2015).

In this study, we first asked the question: “Is one more likely to observe wildlife from one road type than another?”. Through the systematic comparison of tar and dirt roads, our results show that the common mammal species in the Kruger National Park are not associated with a specific road type. One is therefore equally likely to observe the common medium‐sized to large herbivores from both tar and dirt roads. In contrast, impala in the Serengeti were found to avoid major roads (Mtui, 2014) and display a sensitivity toward higher traffic densities associated with main roads (Lunde et al., 2016). Mtui (2014) selected roads varying in traffic volume for her study, but does not mention actual traffic volume numbers. It is possible that the difference in traffic volume between the major, busy roads in the Serengeti and the quieter roads was larger in her study than in the Kruger Park. Although we found a five times higher traffic volume on the tar roads of the park than its dirt roads, this is still far lower than that of large national roads (Jackson et al., 2017) and is therefore likely not high enough to deter animals from tar roads. Thus, the threshold traffic intensity at which animals avoid roads (Jackson et al., 2017) is probably not reached in the Kruger Park, especially given the relatively low speed limit of 50 km/h. Our results also contradict the findings, specifically for impala, of Mulero‐Pázmány et al. (2016), who reported twice as many impala observations per 10 km stretch on dirt roads, compared to tar roads, resulting in conclusions that suggested avoidance of tar roads by this species. These conclusions were, however, contested by Jackson et al. (2017), who, using the same dataset, found no robust support for such avoidance. Our study did not assess drivers of habitat selection of the study species, but the lack of differences in encounters between road surface types suggests that other local factors (e.g., forage quality, predator avoidance, and social interactions) likely supersede any effects on distribution associated with road types and traffic volumes.

Although our results show that the likelihood of observing game is equal from tar and dirt roads, it is possible that typically observed herd sizes differ between road types as a result of road‐specific road avoidance. Our findings, however, show that the likelihood of observing larger herd sizes does not differ between tar and dirt roads for the three most often‐observed species, elephant, impala, and zebra. Thus, one is equally likely to see large herds of these species from either road type. This result is likely associated with broadscale habituation of species to human infrastructure and vehicle traffic in Kruger National Park (Jackson et al., 2017; Mason, 1990; Mills et al., 2004). However, our study was limited to winter months (dry season), a time of year when herd sizes of all three species tend to be smaller (Jarman & Jarman, 1973; Klingel, 1969; Western & Lindsay, 1984). Repeated surveys during other times of the year, particularly austral spring and summer (the wet season), are needed to determine whether changes in social structure that result in larger herd sizes may lead to any road‐type‐associated effects on herd sizes not detected in this study.

Lastly, we explored the hypothesis that the distance of sightings from the road differs between road types and, specifically, that animals should be observed closer to dirt than tar roads, as a result of expected increased avoidance of tar roads compared with dirt roads. Our results show that impala and zebra occur equally often close to tar and dirt roads, again contradicting Mulero‐Pázmány et al. (2016), who suggested that impala were unaffected by dirt roads, but avoided close proximity (at least 10 m) to tar roads. It is noteworthy that we recorded during the day when we could see properly, but that the results may differ at night, when traffic intensities are greatly reduced and herbivores tend to move closer to roads in general (Ager et al., 2003; Eldegard et al., 2012).

For elephant, our results suggest that closer sightings are more likely next to tar, compared to dirt roads, that is, the opposite what we hypothesized. We selected the boundary between the two distance categories conservatively at 20 m, a distance that we could confidently identify in the field. Given that the area covered by the close category (0–20 m) was smaller than that covered by the far category (20 m and beyond), the likelihood of observing animals in the far category was larger than for the close category. Thus, our observation of an increased likelihood of close elephant sightings next to tar roads is even more surprising and indicates preferential use of areas in close proximity to tar roads.

Although we did not assess potential reasons to explain the difference in elephant observation distances between road types, we suggest that this is linked to altered vegetation structure, and, more specifically, an increase in woody canopy cover on the edges of the park roads, compared to areas further away (Smit & Asner, 2012). This difference—which is detectable up to 15 m from the road edge, but is most prominent within the first 5 m of the roadside—has been attributed to altered hydrological regimes next to roads (Smit & Asner, 2012), as roads deliver excess runoff rainwater to road verges (Trombulak & Frissell, 2000). We suggest that this effect is more pronounced for the often wider and less permeable tar roads of the park than its dirt roads. Thus, we suggest that tar roads generate more runoff, resulting in greater increases in woody cover for tar road verges than dirt road verges. Although elephants can adapt to a wide variety of habitats (Roever et al., 2012), tree cover is important for habitat selection in the dry season (Roever et al., 2012) and elephants would therefore be attracted to this increase in woody cover next to tar, compared to dirt roads. Thus, our results show that, in some cases, tar roads can provide benefits to some species.

In general, our finding that all seven commonly occurring species were occasionally found right on the road (0 m distance), for both road types, shows that, at present traffic volumes, the roads of the park do not create a barrier effect for these species. It supports the notion that many of the park's species are habituated to human infrastructure and road disturbance (Jackson et al., 2017; Mason, 1990; Mills et al., 2004), but that the extent of this may be species‐specific. For example, almost a third of all warthog observations were made on the road (results not shown). Although warthog were the smallest of the often‐observed species, making them more difficult to see in the vegetation, it is also possible that the unobstructed road surface provides easy traveling conditions (May & Norton, 1996) for this species.

In protected areas, especially areas reliant on tourism as an income, such as the Kruger National Park, a balance between ecosystem protection and tourist satisfaction needs to be found. The equal likelihood of wildlife viewing, including an equal likelihood of viewing large herd sizes, from tar and dirt roads, has implications for both park management and tourists. For example, temporary closures of dirt roads for rehabilitation and maintenance, which are often required due to the increased runoff leading to erosion (Spies et al., 2018), need not detract from the overall game viewing perspective for most tourists and is something that can also be communicated to park visitors. Our results suggest that tourists do not need to make use of dirt roads, which are often less accessible without high clearance vehicles, to view the species in our study. In fact, close‐up encounters with elephant seem more likely next to tar than dirt roads. However, annual visitor numbers to the Kruger National Park are high, approaching 2 million visitors per year during our study period (South African National Parks, 2018, 2019, 2020) and this number seems to be increasing over time (e.g., South African National Parks, 2008, 2009). As a result, congestion on the park roads leads to visitor complaints (Ferreira & Harmse, 2014). We therefore suggest that any information on animal road‐type associations is conveyed cautiously, so that a balance is struck between encouraging tar road use to conserve the park's more vulnerable dirt roads and avoiding even further congestion on the park's busier tar roads.

## CONFLICT OF INTERESTS

None declared.

## AUTHOR CONTRIBUTIONS

**Misha Malherbe:** Conceptualization (supporting); data curation (lead); formal analysis (lead); investigation (equal); visualization (lead); writing‐original draft (lead). **Trevor McIntyre:** Conceptualization (supporting); formal analysis (supporting); Methodology (equal); writing‐original draft (supporting). **Tarryn V. Hattingh:** Conceptualization (supporting); investigation (equal). **Paige M. Leresche:** Conceptualization (supporting); investigation (equal). **Natalie S. Haussmann:** Conceptualization (lead); funding acquisition (lead); methodology (equal); project administration (lead); supervision (lead); writing‐original draft (supporting).

## Data Availability

The full dataset of sightings recorded through this project was uploaded to the Dryad data repository (https://doi.org/10.5061/dryad.bcc2fqzd1).
